# A benchmark for RNA-seq quantification pipelines

**DOI:** 10.1186/s13059-016-0940-1

**Published:** 2016-04-23

**Authors:** Mingxiang Teng, Michael I. Love, Carrie A. Davis, Sarah Djebali, Alexander Dobin, Brenton R. Graveley, Sheng Li, Christopher E. Mason, Sara Olson, Dmitri Pervouchine, Cricket A. Sloan, Xintao Wei, Lijun Zhan, Rafael A. Irizarry

**Affiliations:** Department of Biostatistics and Computational Biology, Dana-Farber Cancer Institute, 450 Brookline Avenue, Boston, MA 02215 USA; Department of Biostatistics, Harvard TH Chan School of Public Health, 677 Huntington Avenue, Boston, MA 02115 USA; Functional Genomics Group, Cold Spring Harbor Laboratory, 1 Bungtown Road, Cold Spring Harbor, NY 11724 USA; Bioinformatics and Genomics Programme, Centre for Genomic Regulation (CRG) and UPF, Doctor Aiguader, 88, Barcelona, 08003 Spain; Department of Genetics and Genome Sciences, Institute for System Genomics, UConn Health Center, Farmington, CT 06030 USA; Department of Physiology and Biophysics, Weill Cornell Medical College, New York, New York USA; Department of Genetics, Stanford University, 300 Pasteur Drive, MC-5477, Stanford, CA 94305 USA; School of Computer Science and Technology, Harbin Institute of Technology, Harbin, China

## Abstract

Obtaining RNA-seq measurements involves a complex data analytical process with a large number of competing algorithms as options. There is much debate about which of these methods provides the best approach. Unfortunately, it is currently difficult to evaluate their performance due in part to a lack of sensitive assessment metrics. We present a series of statistical summaries and plots to evaluate the performance in terms of specificity and sensitivity, available as a R/Bioconductor package (http://bioconductor.org/packages/rnaseqcomp). Using two independent datasets, we assessed seven competing pipelines. Performance was generally poor, with two methods clearly underperforming and RSEM slightly outperforming the rest.

**Electronic supplementary material:**

The online version of this article (doi:10.1186/s13059-016-0940-1) contains supplementary material, which is available to authorized users.

## Background

RNA sequencing (RNA-seq) has become one of the most widely used technologies in biomedical research for highly parallel measurements of transcript expression. For example, the ENCODE project is currently using RNA-seq to characterize the transcriptome of the project’s selection of cell lines [1]. The first step in quantifying transcription levels with RNA-seq is aligning reads, or pseudo-aligning parts of the read [2, 3], to transcripts. In this step, transcripts are either estimated from the data (de novo assembly) or predetermined from an existing database. In a second step, the expression level for each of the transcripts in consideration is quantified for each sample. The algorithms considered here quantify expression for alternative transcripts within each gene and these can be combined to provide a summary for genes. We will refer to these two outputs as *transcript level* and *gene level*, respectively.

Currently, there are several competing algorithms for both of these steps. In general, when a new method is published, authors typically claim superiority over existing methods. This results in contradictory information for those deciding on a method. This apparent contradiction is due to the lack of a predetermined standard for comparison, which gives authors the freedom to select evaluation procedures that favor their method. This phenomenon is known as the *self-assessment trap* [4]. To avoid this, one can define metrics beforehand that evaluate specificity/precision and sensitivity/accuracy. A previous study [5] implemented such an approach and evaluated 11 algorithms. All algorithms were found to perform remarkably well and none were reported to outperform or underperform. However, accuracy assessments were related to the quantification of absolute expression levels, yet most RNA-seq studies are interested in relative measures, or differential expression. Furthermore, the specificity assessment was based on the correlation of measurements from replicated experiments, a summary that we show to be suboptimal. Finally, most of the assessment was based on computer-simulated data that do not mimic experimental data in sources of variation, such as batch effects [6]. Here we contribute a new set of interpretable assessment metrics, motivated by previous work [7–9], that (1) relate to differential expression, (2) provide improvements over the use of correlation by considering direct estimates of variance, and (3) are based on data that better emulate experimental data. This set of assessments better discerns the differences between the competing algorithms. To demonstrate the utility of our assessment metrics, we use them to compare the STAR [10], TopHat2 [11], and Bowtie2 [12] mapping methods and the Cufflinks [13], eXpress [14], Flux Capacitor [15], kallisto [2], RSEM [16], Sailfish [3], and Salmon [17] quantification methods. We also developed a webtool (http://rafalab.rc.fas.harvard.edu/rnaseqbenchmark) that permits users to submit other competing methods.

## Results

### Datasets

A key aspect of our proposed assessment is the availability of two datasets that permit the computation of the metrics. They are characterized by including two populations, at least two replicates for each population, and a way to define beforehand which genes or transcripts are differentially expressed and which are not. The replicates permit the assessment of precision and comparing the two populations permits the assessment of sensitivity or the ability to discover real biological differences. Note that sensitivity is harder to assess because we need to know beforehand what differences to expect. In the past, this has been achieved with spike-in experiments [18–20]. However, the use of spike-ins has been criticized for not properly mimicking real experimental data [21].

The first represents the minimal dataset that can be used for the comparison. It includes two replicates for the cell lines GM12878 and K562. We used results from a microarray experiment comparing the same two cell lines to define real biological differences. We defined genes with a q-value smaller than 0.05 [22, 23] and absolute log fold changes larger than 1 as truly differentially expressed. Genes with q-values larger than 0.5 were denoted as not differentially expressed. Genes in neither of these two groups were considered to be in a “grey area” and left out of the analysis. Note that we are not considering microarrays to be a gold standard, but because the microarray data represents an independent measurement, algorithms that perform better at detecting real differences should, on average, show improved agreement with these independent results.

**Fig. 1:**
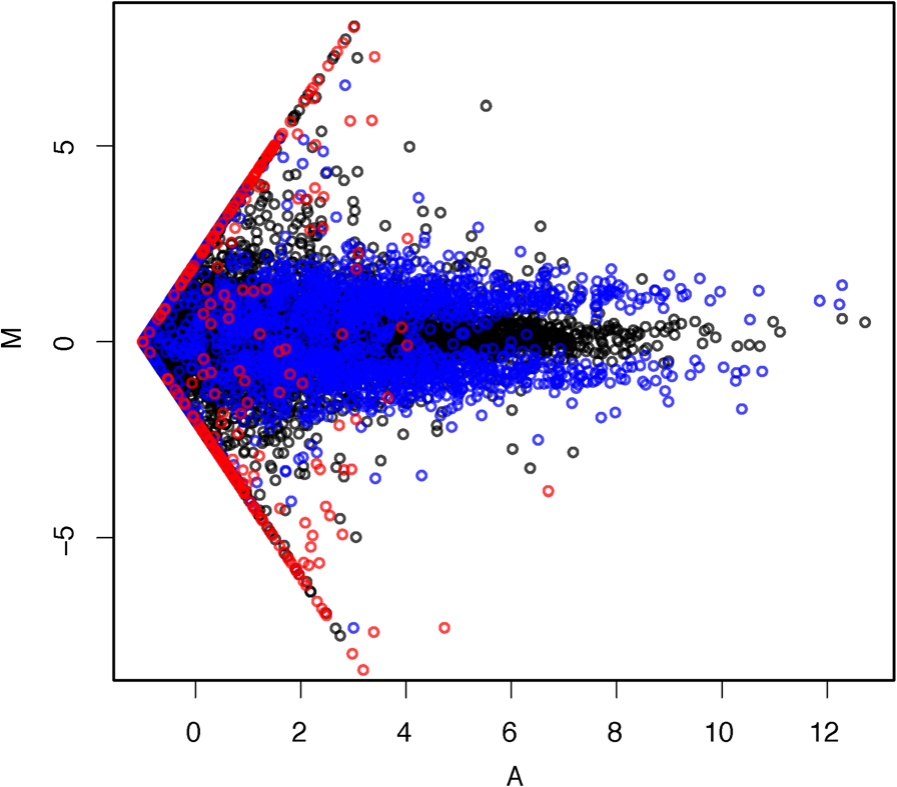
Estimated log fold changes stratified by transcript abundance on simulation dataset. One example based on Cufflinks quantification of two samples is shown here. *Black points* are non-differential transcripts; *blue points* are differentially expressed transcripts which were simulated to have signals on both samples; *red points* are differentially expressed transcripts which were simulated to have signals in only one of the samples

The second dataset was created using 30 samples from the Geuvadis project [24]. These samples were selected to represent a random sample of individuals. To introduce batch effect-like variability, we selected 15 from one center and 15 from another. These were then randomly divided into two groups both having seven samples from one center and eight from another. Because the samples were assigned at random, this is a null experiment and we can consider the 15 samples in each group to be replicates. To distinguish the two groups, we used computer simulations to generate 2424 transcripts designed to be differentially expressed between the two groups. To make these abundances mimic experimental data, we adapted the Polyester method [25] to include GC bias imitating the bias observed in the actual data. The resulting dataset mimics a real one quite well (Fig. 1).

The raw sequencing files for both datasets are available from the webtool (http://rafalab.rc.fas.harvard.edu/rnaseqbenchmark). Further details about both datasets are available in the “Methods” section.

### Calibration using control genes

**Fig. 2:**
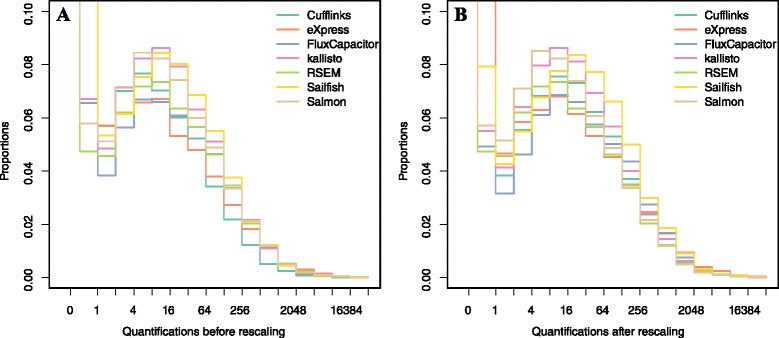
Distribution of reported transcript quantifications on one sample of simulation dataset **a** before and **b** after rescaling. Seven quantification methods are shown here

The first challenge to comparing performance across the different approaches is the lack of a standard unit for transcript level quantification. For example, Cufflinks reports fragments per kilobase of exon per million fragments mapped (FPKM); Flux Capacitor reports reads per kilobase of exon per million reads mapped (RPKM); eXpress, RSEM, Sailfish, kallisto, and Salmon report transcripts per million (TPM). Note that some of these algorithms provide options for what unit to return and we allowed each laboratory to decide which unit to report. Other analytical choices, such as the choice of normalization approach, add even more variability to the scales of the reported measures (Fig. 2a). The standard solutions to rescaling—for example, dividing by the median—are not appropriate because the median value for a typical sample is 0. Taking the median of the positive values is not appropriate as it may introduce a bias in favor of or against methods that over- or underestimate the number of features with no expression. To overcome these challenges we considered only genes reported to be house-keeping genes [26], which are more likely to be expressed. Specifically, considering only this subset of genes for each algorithm, we compute the median. Because house-keeping genes are typically expressed, this median value will not be 0. We then select one of the algorithms to serve as a reference baseline (we used RSEM, which reported in TPM) and we rescale all methods in the log-scale so that a value of 0 in the log-scale maps to a TPM of 1. Figure 2 shows the data before and after this rescaling. Note that this is not meant as a normalization step, but rather as a simple rescaling so that the reported quantifications are approximately in the same units for all methods. Furthermore, note that fold change values are not affected by this re-scaling since the measurements for each algorithm are divided by the same constant which is cancelled out in fold-change calculations.

### Correlation is not a measure of precision or reproducibility

The use of correlation to summarize reproducibility has become widespread in genomics [18, 27, 28]. Despite its English language definition, mathematically, correlation is not necessarily informative with regards to reproducibility. For this reason, we dedicate a subsection to explain the major problems with this metric (details are provided in the “Methods” section).

The most egregious related mistake is to compute correlations of raw FPKM, RPKM, or TPM data. Averages, standard deviations, and correlations are popular summary statistics for two-dimensional data because, for the bivariate normal distribution, these five parameters fully describe the distribution [29]. However, RNA-seq data are not well approximated by bivariate normal data (Fig. 2). In fact, these data have a very large right tail, which implies that the correlation estimate can be highly susceptible to one point (Additional file 1: Figure S1a). Using the log transformation is a way to ameliorate this problem.

The standard way to quantify reproducibility between two sets of replicated measurements, say *X*_1_ … *X*_*N*_ and *Y*_1_ … *Y*_*N*_, is simply to determine how close they are to each other. To quantify distance, we compute the mathematical distance between them:

$$ \sqrt{\varSigma_{i=1}^N\kern0.5em {d}_i^2,}\kern2em \mathrm{with}\kern0.5em {d}_i={X}_i-{Y}_i $$

This metric decreases as reproducibility improves and is 0 when the reproducibility is perfect.

Another limitation with correlation is that it is defined from two lists and there is no standard way of applying it when more than one replicate is available. A standard measure of precision, the standard deviation (SD) across replicates, is more appropriate. Note that there is a connection between this metric and distance since the SD for two replicates is:

$$ \sqrt{\frac{1}{2}\left\{{\left({X}_i\kern0.5em -\kern0.5em \frac{X_i\kern0.5em +\kern0.5em {Y}_i}{2}\right)}^2\kern0.5em +\kern0.5em {\left({Y}_i\kern0.5em -\kern0.5em \frac{X_i\kern0.5em +\kern0.5em {Y}_i}{2}\right)}^2\right\}}\kern0.5em =\kern0.5em \frac{X_i\kern0.5em -\kern0.5em {Y}_i}{2}\kern0.5em =\kern0.5em \frac{d_i}{2} $$

Another limitation of the correlation is that it does not detect cases that are not reproducible due to average changes. These could happen, for example, if the data are not properly normalized. The distance metric does detect these differences (Additional file 1: Figs. S1b and S2). The mathematical details are provided in the “Methods”.

Another advantage of the SD metric is that it is in the same units as our measurements. Correlation lacks units and thus renders the metric hard to interpret. In the “Methods” section, we mathematically demonstrate that correlations near 1 do not necessarily imply reproducibility. Specifically, we show an equation explaining how we may encounter situations in which the distance between two measures is unacceptably high yet correlations close to 1 are achieved.

### Precision metrics

Once the raw data are mapped, quantified, and re-scaled, a matrix with one column for each replicate and thousands of rows is produced for each group. The entries of this matrix are what the algorithms provide as a measure that is proportional to expression. Here we will denote this quantity with ${Y}_{\mathit{\mathsf{g}}ij}$ (where $\mathit{\mathsf{g}}\kern0.5em =\kern0.5em  1\kern0.5em \dots \kern0.5em G$) being the feature (gene or transcript), *i* = 1, 2 identifying the two groups and *j* = 1 … *J*_*i*_ representing the replicate within group *i*.

Our first metric is based on the standard deviations, denoted *s*_*gi*_, of $\log \left({Y}_{gi1}+0.5\right),\dots, \log \left({Y}_{gi{J}_i}+0.5\right)$. This metric has an intuitive interpretation as it represents the typical log fold change observed when comparing expression values from replicate samples. We compute the SD on the log-scale because biologists quantify differential expression with fold changes. Because the log is not defined when the *Y*_*gij*_ values are 0, we add the constant 0.5 [30] before computing the log. In the case of two replicates, the SD would be proportional to the absolute value of the log ratios:

*M*_*gi*_ = log_2_{(*Y*_*gi*1_ + 0.5)/(*Y*_*gi*2_ + 0.5)}

Note that we have an *s*_*gi*_ for every transcript and each group. To provide one summary, we can average across all the features to obtain one measure of reproducibility:

$$ \sqrt{\frac{1}{2}\left(\frac{1}{G}{\displaystyle \sum_{g=1}^G}{s}_{g1}^2+\frac{1}{G}{\displaystyle \sum_{g=1}^G}{s}_{g2}^2\right)} $$

For the case of two replicates this is proportional to Euclidean distance. The smaller this quantity, the more reproducible we assess the algorithm to be. However, as we describe below, providing just one summary is simplistic due to the dependence of variability on abundance.

Empirical and theoretical evidence suggests that the range of *s*_*gi*_ is larger for lower abundance transcript measurements; thus, visualization approaches plot *s*_*i*_ versus a measure of average abundance:

$$ {A}_{gi}=1/{J}_i{\displaystyle \sum_{j=1}^{J_i}}\left({ \log}_2{Y}_{gij}+0.5\right) $$

**Fig. 3:**
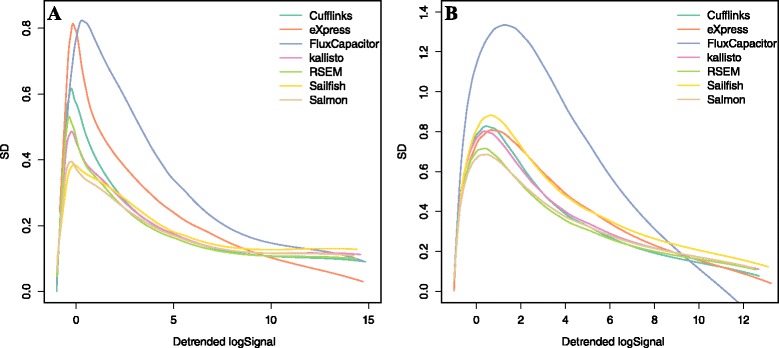
Standard deviations of transcript quantifications based on **a** an experimental dataset (GM12878) and **b** a simulation dataset (one of the cell lines). Seven quantification methods are shown here

Additional file 1: Figure S3a confirms that larger variability is observed for smaller values of *A*. Consider that we may prefer a method that outperforms for the values of *A* that are most common (for example, note that less than 45 % of the data has *A* values larger than 2). Now, this plot does not lend itself to visualizations that permit comparisons across methods, as each method needs its own plot. To create a reasonable summary plot that includes all the methods, we simplify the relevant information by estimating s_*i*_ as a function of *A*_*i*_ [31]. Specifically, we apply loess [32] to estimate this function. We can then plot several curves on the same plot to compare methods (Fig. 3). To provide summary statistics that take into account the dependence on abundance, we report the median *s*_*i*_ for low (A lower than 1), medium (A between 1 and 6), and high (A larger than 6) strata (columns 2–4 in Tables 1 and 2). We include standard error estimates of these metrics as well.

**Table 1:** Summarized metrics for analyzed pipelines based on an experimental dataset

Method	SD low	SD medium	SD high	NE (K = 1)	NN (K = 1)	TxDiff low	TxDiff medium	TxDiff high	deFC low	deFC medium	deFC high	pAUC
Cufflinks	0.62 (0.002)	0.26 (0.001)	0.12 (0.000)	0.08	0.70	0.31 (0.007)	0.08 (0.002)	0.03 (0.001)	2.65 (0.022)	2.25 (0.047)	1.01 (0.024)	0.77
eXpress	0.75 (0.003)	0.37 (0.002)	0.13 (0.001)	0.05	0.80	0.44 (0.008)	0.05 (0.002)	0.01 (0.000)	1.93 (0.026)	2.56 (0.058)	1.20 (0.028)	0.68
Flux Capacitor	0.62 (0.003)	0.57 (0.003)	0.18 (0.001)	0.10	0.73	0.42 (0.008)	0.15 (0.004)	0.07 (0.003)	2.62 (0.024)	2.40 (0.050)	1.01 (0.025)	0.75
kallisto	0.53 (0.002)	0.24 (0.001)	0.12 (0.000)	0.09	0.64	0.28 (0.007)	0.08 (0.002)	0.03 (0.0001	2.36 (0.024)	2.06 (0.045)	1.03 (0.024)	0.76
RSEM	0.54 (0.002)	0.22 (0.001)	0.11 (0.000)	0.06	0.73	0.39 (0.008)	0.07 (0.002)	0.02 (0.001)	2.72 (0.022)	2.22 (0.048)	1.03 (0.026)	0.78
Sailfish	0.46 (0.002)	0.25 (0.001)	0.13 (0.000)	0.08	0.60	0.27 (0.006)	0.08 (0.002)	0.04 (0.001)	2.30 (0.023)	2.08 (0.044)	0.97 (0.022)	0.77
Salmon	0.46 (0.002)	0.23 (0.001)	0.12 (0.000)	0.08	0.65	0.29 (0.007)	0.07 (0.002)	0.04 (0.001)	2.30 (0.024)	2.06 (0.045)	1.03 (0.022)	0.77

Metrics for single cell lines are averaged for both cell lines, except standard deviation is the square root of average squares. Columns 2–4 shows median standard deviation on three transcript abundance levels; column 5 shows proportions of discordant calls when K = 1; column 6 shows proportions of both non-expressed when K = 1; columns 7–9 show the mean proportion differences of transcripts in genes only having two annotated transcripts based on three transcript abundance levels; columns 10–12 show median log fold changes of true differentially expressed genes based on three abundance levels; column 13 shows standardized partial area under the curve for differential expression of genes. *pAUC* partial area under the receiver operating characteristic curve

**Table 2:** Summarized metrics for analyzed pipelines based on a simulation dataset

Method	SD low	SD medium	SD high	NE (K = 1)	NN (K = 1)	TxDiff low	TxDiff medium	TxDiff high	deFC low	deFC medium	deFC high	pAUC
Cufflinks	0.73 (0.002)	0.54 (0.003)	0.26 (0.001)	0.090	0.657	0.34 (0.011)	0.08 (0.003)	0.05 (0.003)	0.53 (0.009)	0.95 (0.006)	0.98 (0.003)	0.61
eXpress	0.71 (0.003)	0.67 (0.004)	0.30 (0.001)	0.09	0.68	0.33 (0.009)	0.07 (0.003)	0.07 (0.003)	0.47 (0.011)	0.87 (0.015)	0.91 (0.012)	0.60
Flux Capacitor	1.03 (0.004)	1.23 (0.007)	0.40 (0.002)	0.15	0.63	0.46 (0.013)	0.15 (0.006)	0.07 (0.004)	0.39 (0.011)	0.82 (0.013)	0.97 (0.009)	0.52
Kallisto	0.72 (0.003)	0.55 (0.003)	0.27 (0.001)	0.10	0.63	0.37 (0.011)	0.08 (0.004)	0.05 (0.003)	0.56 (0.008)	0.95 (0.006)	0.98 (0.002)	0.58
RSEM	0.65 (0.002)	0.48 (0.003)	0.25 (0.001)	0.08	0.69	0.43 (0.011)	0.07 (0.004)	0.04 (0.003)	0.58 (0.008)	0.96 (0.006)	1.00 (0.003)	0.65
Sailfish	0.76 (0.003)	0.65 (0.004)	0.30 (0.001)	0.11	0.57	0.34 (0.009)	0.08 (0.004)	0.05 (0.003)	0.52 (0.011)	0.94 (0.011)	0.96 (0.006)	0.56
Salmon	0.64 (0.002)	0.52 (0.003)	0.26 (0.001)	0.09	0.67	0.35 (0.010)	0.08 (0.004)	0.05 (0.003)	0.54 (0.008)	0.95 (0.007)	1.00 (0.003)	0.61

The last four columns are based on differential expression of transcripts. *pAUC* partial area under the receiver operating characteristic curve

In the first dataset, Flux Capacitor and eXpress clearly underperform compared with the other methods in the regions with most data (*A* between 3 and 8). In the second dataset, only Flux Capacitor clearly underperforms. The other methods performed similarly, with RSEM performing slightly better in both datasets. Overall, the precision was substantially worse than what we observe for microarrays (compare, for example, with Fig. 2 in [31]). This is particularly true for low abundance transcripts where even the best methods show a standard deviation of 0.5, which translates to a difference of 41 % between replicate measurements.

Although we observed differences between quantification algorithms, different aligners show similar results (Additional file 1: Figure S4). STAR generally outperformed TopHat2, although very marginally. Also, RSEM mapped with Bowtie2 outperformed RSEM with STAR (Additional file 1: Figure S4a).

**Fig. 4:**
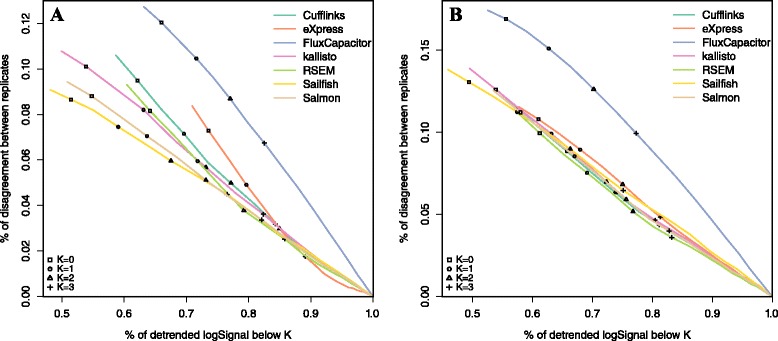
Proportions of discordant expression calls based on **a** an experimental dataset (GM12878) and **b** a simulation dataset (one of the cell lines). Seven quantification methods are shown here

Because a large percentage of the quantifications are 0, we also developed summary statistics and visualization techniques to assess the across-replicate consistency of 0 calls. Note that the features considered here are those with at least one 0 in the pair of replicated measures. For each of these we report the proportion of discordant calls:

$$ {D}_i(K)= \Pr \left[\left({Y}_{gi1}<K\ \&{Y}_{gi2}\ge K\right)\mathrm{or}\ \left({Y}_{gi1}\ge K\ \&\ {Y}_{gi2}<K\right)\right] $$

where *K* is a threshold defining a transcript as expressed. Because methods that call more 0s are less likely to be discordant, we plot *D*_*i*_(*K*) against the proportion of the transcript (Fig. 4). The results here are similar to those of Fig. 3. We report *D*_*i*_(*K*) for *K* = 1 in column 5 of Tables 1 and 2. In both datasets we see Flux Capacitor as clearly underperforming compared with the other methods.

### Consistency of isoform calls

**Fig. 5:**
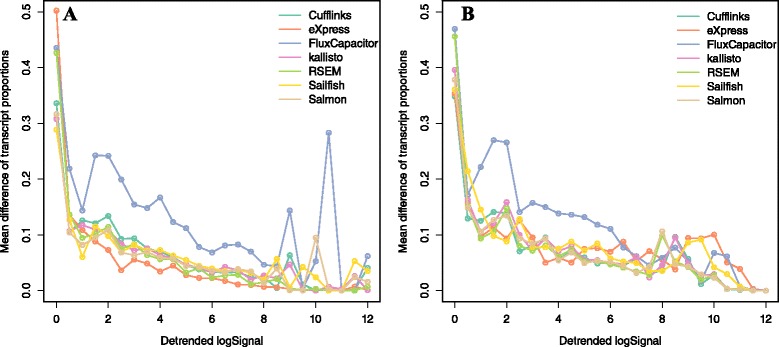
Proportion differences of transcript quantifications in genes with only two annotated transcripts based on **a** an experimental dataset (GM12878) and **b** a simulation dataset (one of the cell lines). Seven quantification methods are shown

Because RNA-seq is commonly used to infer alternative transcription, we also assessed the reproducibility of abundance within transcripts of the same gene. To provide a simple and interpretable metric we considered only the genes with exactly two transcripts. Specifically, for each sample we computed the percentage of reported abundances for each of the first transcripts. So if *t*_*1*_ and *t*_*2*_ are the reported abundances for transcripts 1 and 2, we compute *t*_*1*_/(*t*_*1*_ + *t*_*2*_). We then performed every pairwise comparison of two replicates and for each gene recorded the difference in these proportions. Note that we expect this difference to be 0 since the same transcripts should be reported in two replicates. We plotted these differences against abundance *A*_*gij*_ since we expected larger differences at lower abundances (Additional file 1: Figure S3b). We then stratified the absolute differences by values of *A*_*gij*_ and computed the median value. This permitted us to compare curves across methods (Fig. 5). Here we found Flux Capacitor to underperform compared with the other methods, which performed similarly. However, it is worth noting how high these values are, especially for low abundance transcripts where the median differences are close to 0.5, meaning that we are basically guessing which transcript is present.

### Sensitivity

The above-described metrics relate to reproducibility or specificity. But given the specificity and sensitivity tradeoff, it is imperative that we also assess sensitivity. For example, a method that calls every gene expressed at 10 TPM has perfect specificity, but would, of course, fail to detect any real differences. Recall that for the first dataset we defined truly differentially expressed genes, not transcripts. For each algorithm, therefore, one measure was constructed for each gene by combining the reported quantities for each transcript using the aggregation method recommended by said algorithm. For the second dataset, defining transcripts that were truly differentially expressed was known by construction.

To assess accuracy, we computed an average log fold change for each of the truly differentially expressed transcripts:

$$ {M}_g=1/{J}_2{\displaystyle \sum_{j=1}^{J_2}}\left({ \log}_2{Y}_{g2j}+0.5\right)-1/{J}_1{\displaystyle \sum_{j=1}^{J_1}}\left({ \log}_2{Y}_{g1j}+0.5\right)\kern0.5em , $$

multiplied it by the sign of the true fold change (so that all true fold changes could be considered positive), and plotted it against the average abundance (Additional file 1: Figure S3c):

$$ {A}_g=1/2{J}_2{\displaystyle \sum_{j=1}^{J_2}}\left({ \log}_2{Y}_{g2j}+0.5\right)+1/2{J}_1{\displaystyle \sum_{j=1}^{J_1}}\left({ \log}_2{Y}_{g1j}+0.5\right) $$

To be able to compare methods, we fitted loess curves to these plots and show the curves for all methods. Note that for the second dataset these curves should be equal to 1 for all values of *A* since all truly differentially expressed transcripts were designed to have true log (base 2) fold changes of 1 (Additional file 1: Figure S5). Here we can see that, with the exception of the underperformance of Flux Capacitor, all methods perform similarly. As we did for the standard deviation metric, we report the median sensitivity measure for three strata of abundance (columns 10–12 in Tables 1 and 2).

### ROC curves and pAUC

**Fig. 6:**
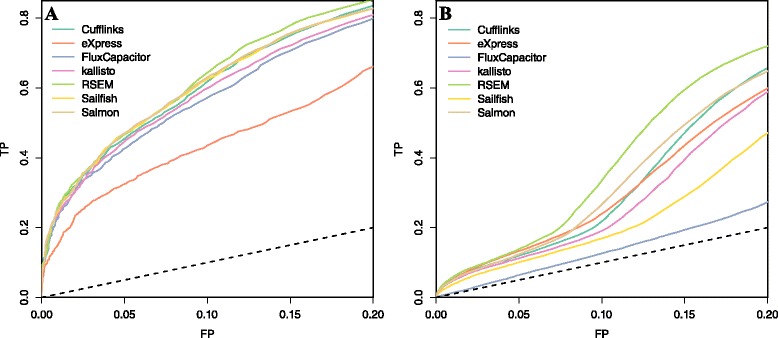
ROC curves indicating performance of quantification methods based on differential expression analysis of **a** an experimental dataset and **b** a simulation dataset. Seven quantification methods are shown. *FP* false positive, *TP* true positive

Finally, to assess sensitivity and specificity simultaneously, we constructed receiver operating characteristic (ROC) curves. Because in the first dataset we used genes and in the second we used transcripts, here we use the general term *feature* to refer to both. We used the same approach as in the previous section to define positives (truly differentially expressed features) and negatives (not differentially expressed features). We then obtained log fold change values for every feature across every pairwise comparison between the two groups. Following common practice, we removed all features with both values below 1. Each of these resulted in one ROC curve. We averaged these results using threshold averaging based on fold changes [33] to produce one ROC curve for each method (Fig. 6). The ROC curves only include false positive rates (FPRs) below 0.2 because, in practice, it would be rare to accept a FPR higher than this since a FPR of 0.2 already represents thousands of false positive features. Here we see Flux Capacitor and eXpress underperforming and RSEM slightly outperforming the other methods in both datasets. The partial (up to FPR of 0.2) area under the curve (pAUC) is included in column 13 of Tables 1 and 2, which is the standardized area under the curve [34].

Although the results for genes are comparable to those seen for microarrays (see Fig. 4 in [8]), we note that the results for transcripts are not impressive in general. For example, to recover half of the real differences, we need to accept a FPR of over 0.15. In fact, to achieve even these results, we removed all transcripts for which both samples were reporting quantities below 1. Without this filtering, the technology does not perform much better than guessing (Additional file 1: Figure S6). This result is in agreement with a recent publication describing the importance of filtering low abundance values in RNA-seq data [35].

## Discussion

Note that for the ROC analysis we show results for both gene level and transcript level analysis and the transcript level metrics were substantially worse (Fig. 6). Previous publications [5] focusing on abundance found that all algorithms performed well. Here we found that if your focus is differential expression, then results are not as impressive and differences are found across algorithms.

We do not intend our study of seven methods to be considered a definitive comparison but rather a demonstration of how one can use simple datasets and interpretable metrics to assess algorithms. For this reason, we have created a webtool that permits the comparison of other methods. Furthermore, we make the software used by this webtool freely available so others can compare methods, including new ones. We note here that the webtool includes a third dataset in which batch effects are completely confounded with group. In the future, this dataset will permit the assessment of methods that adjust for batch. An important contribution is that we have fixed assessments, making it harder for developers to fall into the self-assessment trap.

Finally, note that our method is meant to assess the quantification method specifically. Because, in general, our method does not consider biological replicates, it is not meant to be used for comparisons of statistical methods such as DESeq2 [36] and edgeR [37].

## Conclusions

We have described a series of metrics and visualization techniques that facilitate the statistical evaluation of algorithms for processing RNA-seq data. The method is applicable with a small experiment involving as few as two sets of replicates and data from an independent platform. Using this approach, we assessed several competing approaches in terms of specificity and sensitivity. With the exception of the underperforming Flux Capacitor and eXpress, we found that the other algorithms performed similarly. We found that overall performance in detecting differentially expressed transcripts was poor. We also found that the mapping algorithms had a comparatively small effect, with STAR slightly outperforming TopHat2 when compared directly.

## Methods

### Cell line data

The dataset used in this comparison was derived from two widely studied cell lines: GM12878 and K562. The RNA-seq data are described by the ENCODE data center (https://www.encodeproject.org/) with dataset accession IDs ENCSR000AED and ENCSR000AEM. Microarray data for these two cell lines were downloaded from GEO with accession ID GSE26312 [38].

### Quasi-simulated dataset

The GENCODE v16 GTF file was downloaded from GENCODE (http://www.gencodegenes.org/) and all protein-coding genes from chromosome 2 with a single isoform or two isoforms were extracted; in addition, 300 genes with 3–15 isoforms were sampled. These genes were used to create a set of transcripts (all the isoforms from the selected genes) from which paired-end reads would be simulated. FPKM values for genes were sampled from the empirical distribution of FPKM values for genes estimated by Cufflinks [13] on the 30 Geuvadis samples, excluding values of FPKM less than 1.8. Expression was distributed randomly to the isoforms within a gene using a flat Dirichlet distribution.

The simulated reads were generated using the Polyester software [25], with a modification to allow for fragment-level GC content bias described below. Paired-end reads were generated assuming an experiment with 30 million reads in total, then scaled down for the simulated samples, which corresponded to Geuvadis samples with lower sequencing depth, and scaled up for the simulated samples corresponding to Geuvadis samples with higher sequencing depth. The scaling was chosen based on the total number of pairs which both aligned using TopHat2 [11]. The fragment length distribution for the simulated paired-end reads was centered at 160 bp and with a SD of 30 bp, chosen to match the fragment width distribution in the 30 Geuvadis samples.

The samples were distributed using a block design with 15 samples in a simulated condition 1 (seven from batch 1 and eight from batch 2) and 15 samples in a simulated condition 2 (eight from batch 1 and seven from batch 2). The bias parameters used to simulate fragment GC bias were drawn directly from the values estimated on the corresponding Geuvadis sample using the alpine software [39]. For 80 % of genes, one of the conditions had the expected mean value multiplied by 2, for 10 % of genes one of the conditions had the expected value equal to 0, and the remaining 10 % of genes represented the null, having no difference in expected value for the mean across conditions.

Fragments generated by Polyester were randomly discarded using Bernoulli trials, with the probability of success given by the estimated dependence of the fragment rate on GC content, as estimated by alpine [39]. As this resulted in less fragments than the original target, the simulation was repeated again, scaling proportionally such that the original target sequencing depth would be reached.

### Transcript annotation

Although the methodology described here can be generally applied to any set of features, for the comparison carried out here we quantified expression levels for transcripts annotated in the GENCODE v16 database [40]. Note that this database includes protein-coding genes, pseudogenes, long non-coding genes (lncRNAs), and others. We focused on protein-coding genes to illustrate our proposed metrics. The units used by the quantification profiles of protein coding genes can be in reads per kilobase of exon per million reads mapped (RPKM), fragments per kilobase of exon per million fragments mapped (FPKM) or transcripts per million (TPM), etc., depending on the pipelines.

### Data quantification and preprocessing

All the RNA-seq samples were first aligned with STAR (version 2.3.1) and Bowtie2 (version 2.2.1). The dataset containing GM12878 and K562 was aligned with TopHat2 (version 2.0.8) as well. Quantification pipelines, including Cufflinks (version 2.2.1), eXpress (version 1.5.1), Flux Capacitor (version 1.5.1), kallisto (version 0.42.3), RSEM (version 1.2.11), Sailfish (version 0.6.2), and Salmon (version 0.5.0) were used to quantify transcript expression levels, represented by units of FPKM, RPKM, or TPM. For more details, such as the commands and parameter settings, refer to the log information on the webtool. RMA [41] was used to normalize microarray samples between two cell lines and corresponding replicates.

### Correlation is not a measure of reproducibility

For lists of numbers to be considered to reproduce another, the differences between the entries of the list must be close to 0. We can summarize with one number by using distance. Note that distance and correlation are related. We can rewrite the distance (squared and divided by *N*) between two vectors *X* and *Y*:

$$ \frac{1}{N}{\displaystyle \sum_{i=1}^N}{\left({X}_i-{Y}_i\right)}^2 $$

as:

$$ \frac{1}{N}{\displaystyle \sum_{i=1}^N}{\left[\left({X}_i-{\mu}_X\right)-\left({Y}_i-{\mu}_Y\right)+\left({\mu}_X-{\mu}_Y\right)\right]}^2 $$

where *μ*_*X*_ is the average of the *X*s and *μ*_*Y*_ is the average of the *Y*s. Then we have:

$$ \frac{1}{N}{\displaystyle \sum_{i=1}^N}{\left({X}_i-{Y}_i\right)}^2=\frac{1}{N}{\displaystyle \sum_{i=1}^N}\left[{\left({X}_i-{\mu}_X\right)}^2+{\left({Y}_i-{\mu}_Y\right)}^2\right]+{\left({\mu}_X-{\mu}_Y\right)}^2-2\frac{1}{N}{\displaystyle \sum_{i=1}^N}\left({X}_i-{\mu}_X\right)\left({Y}_i-{\mu}_Y\right) $$

Note that the last term is the covariance. To simplify this equation assume that *X* and *Y* have been standardized to have standard deviation 1. Then the equation reduces to:

$$ \frac{1}{N}{\displaystyle \sum_{i=1}^N}{\left({X}_i-{Y}_i\right)}^2=2+{\left({\mu}_X-{\mu}_Y\right)}^2-2\cdot \mathrm{correlation} $$

and we see the direct relationship between distance and correlation. However, an important difference is that the distance contains the term (*μ*_*X*_ − *μ*_*Y*_)^2^ and can therefore detect cases that are not reproducible due to large average changes. These could happen, for example, if the data are not properly normalized.

The above calculation can be re-expressed in a way that shows yet another flaw with correlation as a measure of reproducibility. Suppose you have a series of measurements *X* and a second measurement differs by *d*. We want the variability of *d* to be as small as possible. However, the correlation between *X* and *X* + *d* can be rewritten as:

$$ \mathrm{corr}\left(X,X+d\right)=\frac{1}{\sqrt{1+\mathrm{v}\mathrm{a}\mathrm{r}(d)/\mathrm{v}\mathrm{a}\mathrm{r}(X)}} $$

which implies that if the variability across values of *X* is very large, as it is in RNA-seq data, you can have correlations close to 1 regardless of the variability of the difference. Note that var(*X*) is about 4 in a typical RNA-seq experiment. This implies that a var(*d*) of 1 results in a correlation of almost 0.9. While 0.9 is considered high by biologists, a variance of 1 is not acceptable as it implies typical across-replicate fold changes of 2.

### Software license

The R/Bioconductor package *rnaseqcomp* is available under open source license GPL-3.

## Additional file

Additional file 1: Figure S1.
**a** A pair of independent normally distributed datasets are simulated. The correlation is 0 by design. The values of one pair were “accidentally” changed to be very large, changing the correlation from 0 to 0.9. **b** For the data in Figure S2, we computed SD, Pearson correlation, and Spearman correlation metrics. A useful metric will detect the first pair (denoted with a *triangle*) as different. Note that the SD metric is by far the best at making this distinction. The Pearson correlation for this pair is above 0.9. **Figure S2.** MA plots for eight pairs of replicates. The first one seems to be problematic as the first replicate is generally larger than the second. The title of each panel shows the SD and demonstrates that this metric does detect the first pair of replicates as being different. **Figure S3.** Three types of proposed metrics change with transcript abundances. Cufflinks quantifications on a simulation dataset are shown here as an example. **a** Standard deviation. **b** Proportion differences of transcripts in genes with only two annotated transcripts. **c** Estimated log fold changes for simulated differentially expressed transcripts. **Figure S4.** Effects of aligners on four major types of metrics. Quantifications for an experimental dataset from cell lines GM12878 and K562 are shown here as an example. Comparison between STAR and TopHat2 are based on Cufflinks and Flux Capacitor. Comparison between Bowtie2 and STAR are based on RSEM. **a** Standard deviations based on the cell line GM12878. **b** Proportion of discordant calls. **c** Proportion differences of transcripts in genes with only two annotated transcripts based on cell line GM12878. **d** ROC curves based on transcript fold changes between GM12878 and K562. **Figure S5.** Log fold changes of true differential expression fitted by loess. **a** Plot based on experimental dataset from cell lines GM12878 and K562. True differentially expressed genes are estimated using microarray data. **b** Plot based on simulation dataset with true differentially expressed transcripts predefined. **Figure S6.** ROC curves on a simulation dataset when no filtering is applied on expression calls. (PDF 1052 kb)
