# Erratum to: A benchmark for RNA-seq quantification pipelines

**DOI:** 10.1186/s13059-016-0986-0

**Published:** 2016-05-23

**Authors:** Mingxiang Teng, Michael I. Love, Carrie A. Davis, Sarah Djebali, Alexander Dobin, Brenton R. Graveley, Sheng Li, Christopher E. Mason, Sara Olson, Dmitri Pervouchine, Cricket A. Sloan, Xintao Wei, Lijun Zhan, Rafael A. Irizarry

**Affiliations:** Department of Biostatistics and Computational Biology, Dana-Farber Cancer Institute, 450 Brookline Avenue, Boston, MA 02215 USA; Department of Biostatistics, Harvard TH Chan School of Public Health, 677 Huntington Avenue, Boston, MA 02115 USA; Functional Genomics Group, Cold Spring Harbor Laboratory, 1 Bungtown Road, Cold Spring Harbor, NY 11724 USA; Bioinformatics and Genomics Programme Centre for Genomic Regulation (CRG) and UPF, Doctor Aiguader, 88, Barcelona, 08003 Spain; Department of Genetics and Genome Sciences, Institute for System Genomics, UConn Health Center, Farmington, CT 06030 USA; Department of Physiology and Biophysics, Weill Cornell Medical College, New York, New York USA; Department of Genetics, Stanford University, 300 Pasteur Drive, MC-5477, Stanford, CA 94305 USA; School of Computer Science and Technology, Harbin Institute of Technology, Harbin, China

After the publication of this work [1] it was noticed that there were typographical errors in the following equations: equation 5 in column 2, equation 7 in column 2, equation 8 in column 1.

The bracket was placed incorrectly, so it should read:

\log_2 (Y_{gij} + 0.5) rather thank (\log_2 Y_{gij} + 0.5)

The publisher apologizes for this error.
