# Erratum to: A benchmark for RNA-seq quantification pipelines

**DOI:** 10.1186/s13059-016-1060-7

**Published:** 2016-09-30

**Authors:** Mingxiang Teng, Michael I. Love, Carrie A. Davis, Sarah Djebali, Alexander Dobin, Brenton R. Graveley, Sheng Li, Christopher E. Mason, Sara Olson, Dmitri Pervouchine, Cricket A. Sloan, Xintao Wei, Lijun Zhan, Rafael A. Irizarry

**Affiliations:** 1 Department of Biostatistics and Computational Biology, Dana-Farber Cancer Institute, 450 Brookline Avenue, Boston, MA 02215 USA; 2 Department of Biostatistics, Harvard TH Chan School of Public Health, 677 Huntington Avenue, Boston, MA 02115 USA; 3 Functional Genomics Group, Cold Spring Harbor Laboratory, 1 Bungtown Road, Cold Spring Harbor, NY 11724 USA; 4 Bioinformatics and Genomics Programme Centre for Genomic Regulation (CRG) and UPF, Doctor Aiguader, 88, Barcelona, 08003 Spain; 5 Department of Genetics and Genome Sciences, Institute for System Genomics, UConn Health Center, Farmington, CT 06030 USA; 6 Department of Physiology and Biophysics, Weill Cornell Medical College, New York, New York USA; 7 Department of Genetics, Stanford University, 300 Pasteur Drive, MC-5477, Stanford, CA 94305 USA; 8 School of Computer Science and Technology, Harbin Institute of Technology, Harbin, China

After the publication of this work [1] it was noticed that there were typographical errors in the following equations: equation 5 in column 2, equation 7 in column 2, equation 8 in column 1.

The bracket was placed incorrectly, so it should read:

\ log _2 (Y_{gij} + 0.5) rather than (\ log _2 Y_{gij} + 0.5)

It was brought to our attention that a new submission to the webtool for the eXpress algorithm for the ENCODE GM12878 dataset performs better than what is reported in the paper. While looking into the reason for this discrepancy we found two errors. First, the commands and parameter settings provided in the log information on the webtool were incorrect. Second, we realized that we ran the eXpress submission differently from the other methods for this particular dataset. One cause for the discrepancy was the accidental use of a different transcript FASTA file. We reran eXpress controlling for these differences and confirmed that better results are attained. Row 2 in Table 1 is changed, and the updated row is below.

**Table 1:** Summarized metrics for analyzed pipelines based on an experimental dataset

Method	SD low	SD medium	SD high	NE (K = 1)	NN (K = 1)	TxDiff low	TxDiff medium	TxDiff high	deFC low	deFC medium	deFC high	pAUC
Cufflinks	0.62 (0.002)	0.26 (0.001)	0.12 (0.000)	0.08	0.70	0.31 (0.007)	0.08 (0.002)	0.03 (0.001)	2.65 (0.022)	2.25 (0.047)	1.01 (0.024)	0.77
eXpress	0.53 (0.002)	0.22 (0.001)	0.10 (0.000)	0.07	0.72	0.24 (0.006)	0.06 (0.002)	0.02 (0.001)	2.86 (0.022)	2.21 (0.048)	1.00 (0.019)	0.79
Flux Capacitor	0.62 (0.003)	0.57 (0.003)	0.18 (0.001)	0.10	0.73	0.42 (0.008)	0.15 (0.004)	0.07 (0.003)	2.62 (0.024)	2.40 (0.050)	1.01 (0.025)	0.75
kallisto	0.53 (0.002)	0.24 (0.001)	0.12 (0.000)	0.09	0.64	0.28 (0.007)	0.08 (0.002)	0.03 (0.0001	2.36 (0.024)	2.06 (0.045)	1.03 (0.024)	0.76
RSEM	0.54 (0.002)	0.22 (0.001)	0.11 (0.000)	0.06	0.73	0.39 (0.008)	0.07 (0.002)	0.02 (0.001)	2.72 (0.022)	2.22 (0.048)	1.03 (0.026)	0.78
Sailfish	0.46 (0.002)	0.25 (0.001)	0.13 (0.000)	0.08	0.60	0.27 (0.006)	0.08 (0.002)	0.04 (0.001)	2.30 (0.023)	2.08 (0.044)	0.97 (0.022)	0.77
Salmon	0.46 (0.002)	0.23 (0.001)	0.12 (0.000)	0.08	0.65	0.29 (0.007)	0.07 (0.002)	0.04 (0.001)	2.30 (0.024)	2.06 (0.045)	1.03 (0.022)	0.77

Metrics for single cell lines are averaged for both cell lines, except standard deviation is the square root of average squares. Columns 2–4 shows median standard deviation on three transcript abundance levels; column 5 shows proportions of discordant calls when K = 1; column 6 shows proportions of both non-expressed when K = 1; columns 7–9 show the mean proportion differences of transcripts in genes only having two annotated transcripts based on three transcript abundance levels; columns 10–12 show median log fold changes of true differentially expressed genes based on three abundance levels; column 13 shows standardized partial area under the curve for differential expression of genes. *pAUC* partial area under the receiver operating characteristic curve

The comparative figures for GM12878 change (panel A Figures 3, 4, 5, 6 and Additional file 1: Figure S5). The new figures are below.

**Fig. 3:**
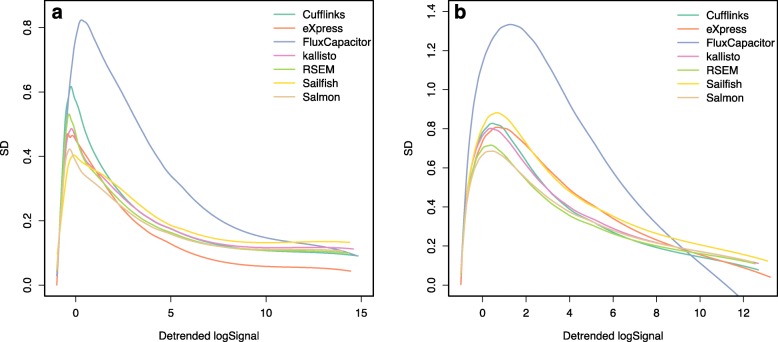
Standard deviations of transcript quantifications based on **a** an experimental dataset (GM12878) and **b** a simulation dataset (one of the cell lines). Seven quantification methods are shown here

**Fig. 4:**
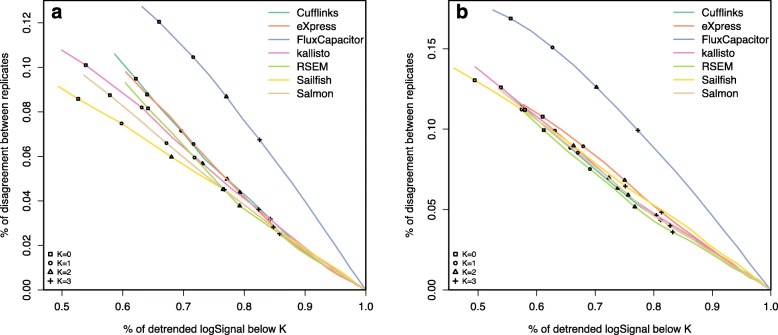
Proportions of discordant expression calls based on **a** an experimental dataset (GM12878) and **b** a simulation dataset (one of the cell lines). Seven quantification methods are shown here

**Fig. 5:**
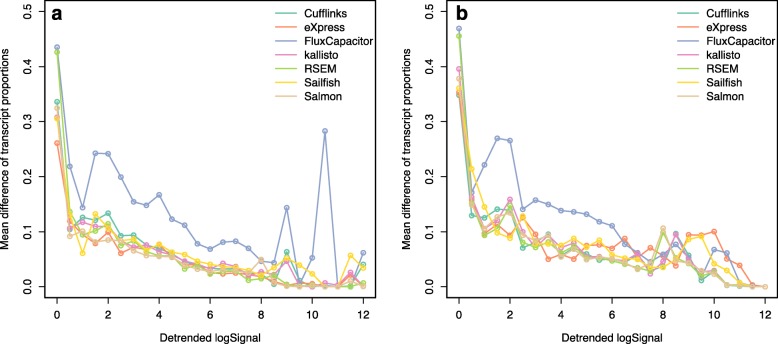
Proportion differences of transcript quantifications in genes with only two annotated transcripts based on **a** an experimental dataset (GM12878) and **b** a simulation dataset (one of the cell lines). Seven quantification methods are shown

**Fig. 6:**
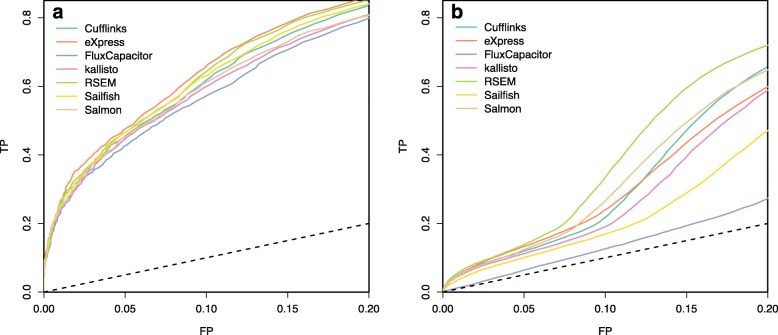
ROC curves indicating performance of quantification methods based on differential expression analysis of **a** an experimental dataset and **b** a simulation dataset. Seven quantification methods are shown. *FP* false positive, *TP* true positive

The following statements should now read:

- *Performance was generally poor, with* ***one*** *method clearly underperforming and RSEM slightly outperforming the rest*.
- *In the first dataset, Flux Capacitor clearly underperform* ***s*** *compared with the other methods in the regions with most data (A between 3 and 8)*.
- *Here we see Flux Capacitor underperforming and RSEM slightly outperforming the other methods in* ***the simulation*** *dataset*.
- *With the exception of the underperforming Flux Capacitor, we found that the other algorithms performed similarly*.

The eXpress entry in the webtool, including the *log-file* entry which includes the scripts, has also been updated. You can see this in the *ENCODE: 2 reps, high depth* tab here: http://rafalab.rc.fas.harvard.edu/rnaseqbenchmark

The authors apologize for this error.

## Additional file

Additional file 1: Figure S5. Log fold changes of true differential expression fitted by losses. (a) Plot based on experimental dataset from cell lines GM12878 and K562. True differentially expressed genes are estimated using microarray data. (b) Plot based on simulation dataset with true differentially expressed transcripts predefined. (PDF 100 kb)
