# Supplementary material for: A benchmark for RNA-seq quantification pipelines
Source: Genome Biol. 2016 Apr 23;17:74. doi: 10.1186/s13059-016-0940-1 (PMC4842274; doi:10.1186/s13059-016-0940-1)
Supplement: Additional file 1: Figure S1. — a A pair of independent normally distributed datasets are simulated. The correlation is 0 by design. The values of one pair were “accidentally” changed to be very large, changing the correlation from 0 to 0.9. b For the data in Figure S2, we computed SD, Pearson correlation, and Spearman correlation metrics. A useful metric will detect the first pair (denoted with a triangle) as different. Note that the SD metric is by far the best at making this distinction. The Pearson correlation for this pair is above 0.9. Figure S2. MA plots for eight pairs of replicates. The first one seems to be problematic as the first replicate is generally larger than the second. The title of each panel shows the SD and demonstrates that this metric does detect the first pair of replicates as being different. Figure S3. Three types of proposed metrics change with transcript abundances. Cufflinks quantifications on a simulation dataset are shown here as an example. a Standard deviation. b Proportion differences of transcripts in genes with only two annotated transcripts. c Estimated log fold changes for simulated differentially expressed transcripts. Figure S4. Effects of aligners on four major types of metrics. Quantifications for an experimental dataset from cell lines GM12878 and K562 are shown here as an example. Comparison between STAR and TopHat2 are based on Cufflinks and Flux Capacitor. Comparison between Bowtie2 and STAR are based on RSEM. a Standard deviations based on the cell line GM12878. b Proportion of discordant calls. c Proportion differences of transcripts in genes with only two annotated transcripts based on cell line GM12878. d ROC curves based on transcript fold changes between GM12878 and K562. Figure S5. Log fold changes of true differential expression fitted by loess. a Plot based on experimental dataset from cell lines GM12878 and K562. True differentially expressed genes are estimated using microarray data. b Plot based on simulation dataset with true differentiall [file 13059_2016_940_MOESM1_ESM.pdf]

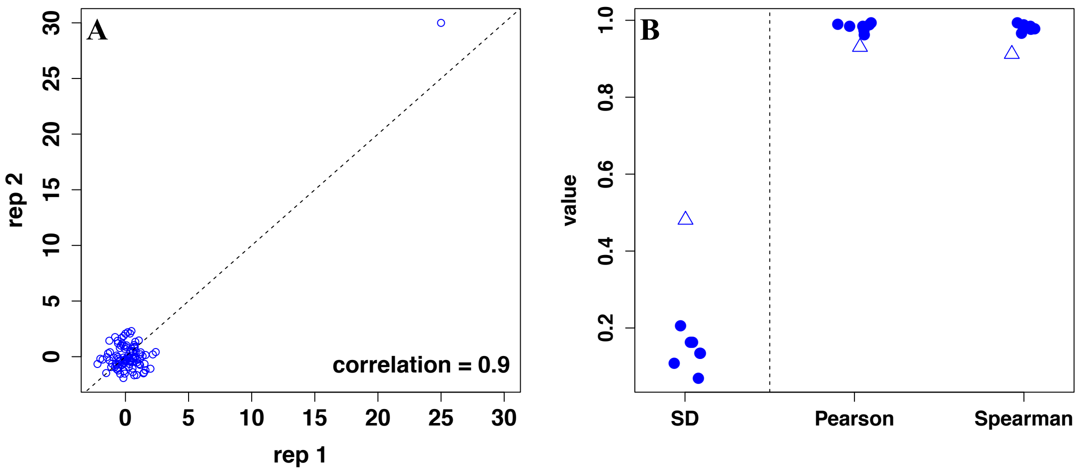


**Figure S1.** (A) A pair of independent normally distributed datasets are simulated. The correlation is 0 by design. The values of one pair as "accidentally" changed to be very large. This one point changes to correlation from 0 to 0.9. (B) For the data in Figure S2, we computed SD, Pearson correlation, and Spearman correlation metrics. A useful metric will detect the first pair (denoted with a triangle) as different. Note that the SD metric is by far the best at making this distinction. The Pearson correlation for this pair is above 0.9.

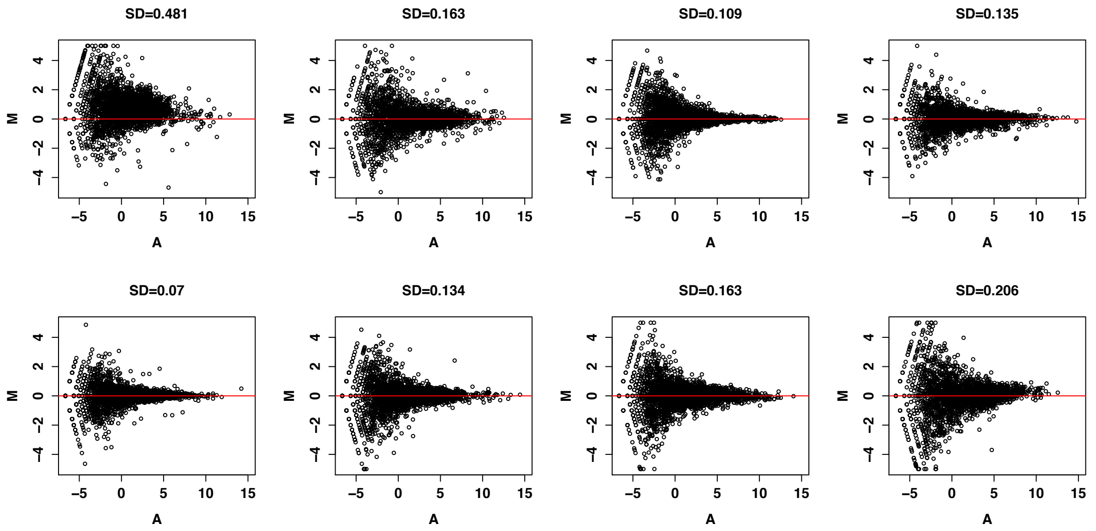


**Figure S2.** MA-plots are shown for eight pairs of replicates. The first one seems to be problematic as the first replicate is generally larger than the second. The title of each pane shows the SD and demonstrates that this metric does detect the first pair of replicates as being different.

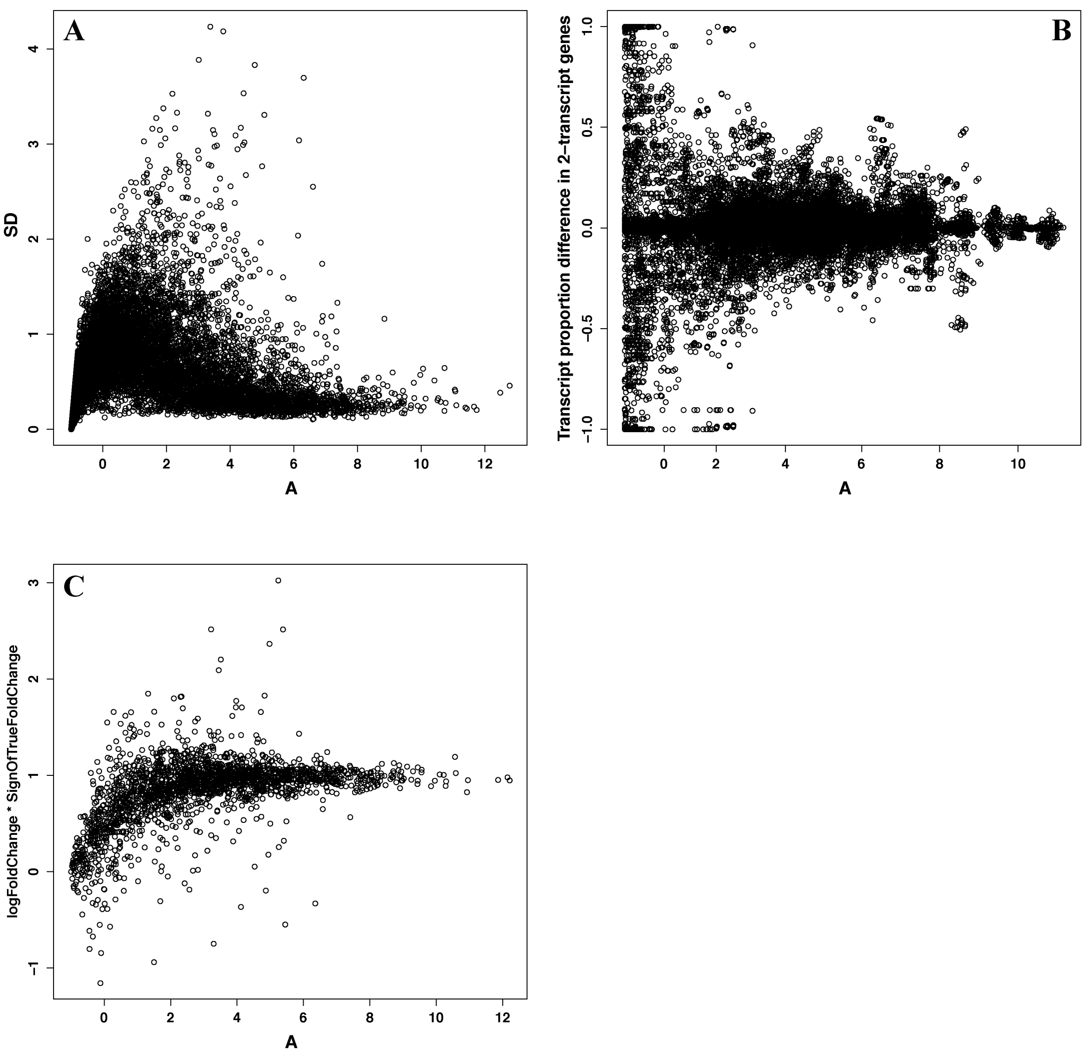


**Figure S3.** Three types of proposed metrics change with transcript abundances. Cufflinks quantifications on simulation dataset are shown here as an example. (A) Standard deviation. (B) Proportion difference of transcripts in genes only have two annotated transcripts. (C) Estimated log fold changes for simulated differential expression transcripts.

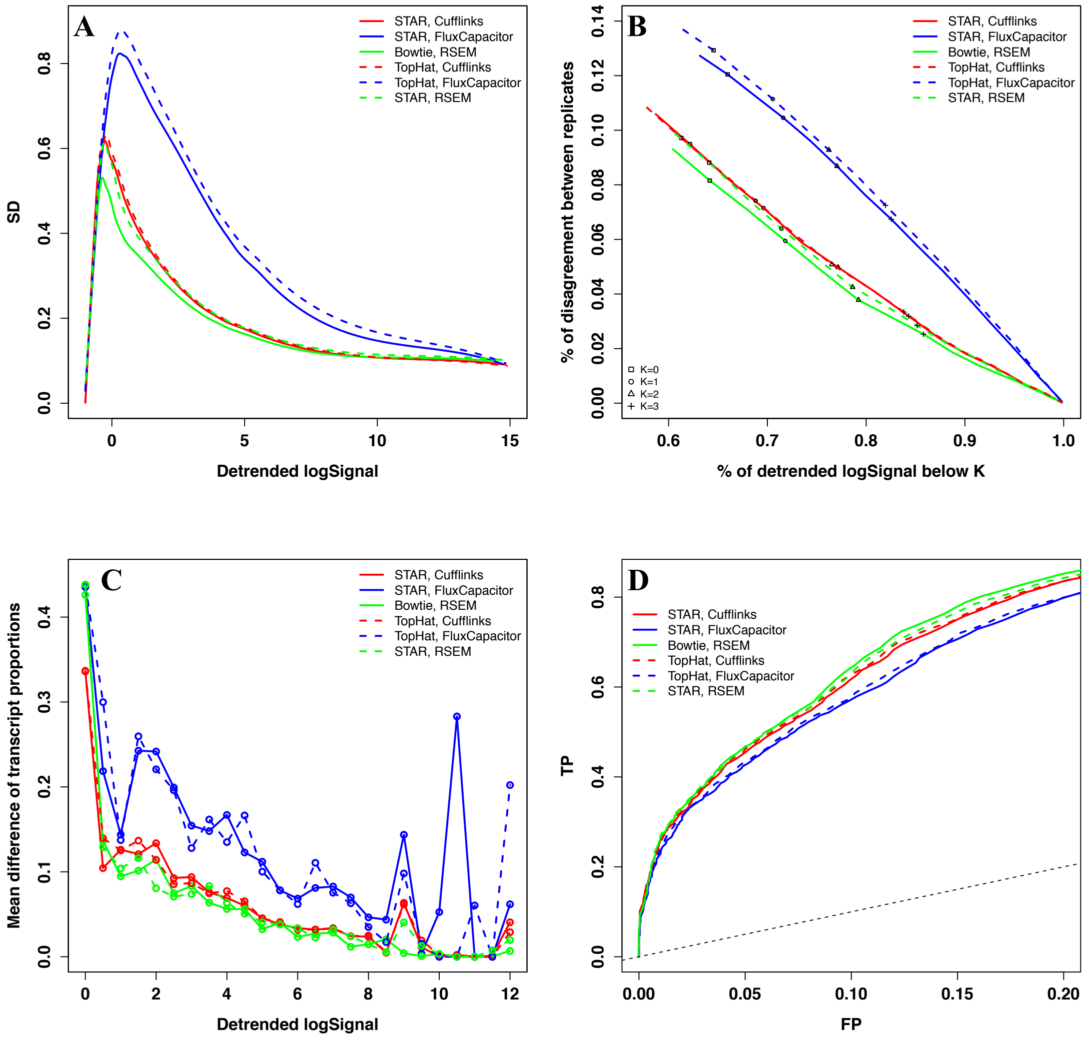


**Figure S4.** Effects of aligners on four major types of metrics. Quantifications on experimental dataset with cell lines GM12878 and K562 are shown here as an example. Comparison between STAR and TopHat2 are based on methods Cufflinks and Flux capacitor. Comparison between Bowtie2 and STAR are based on RSEM. (A) Standard deviations based on cell line GM12878. (B) Proportion of discordant calls. (C) Proportion differences of transcripts in genes only have two annotated transcripts based on cell line GM12878. (D) ROC curves based on transcript fold changes between GM12878 and K562.

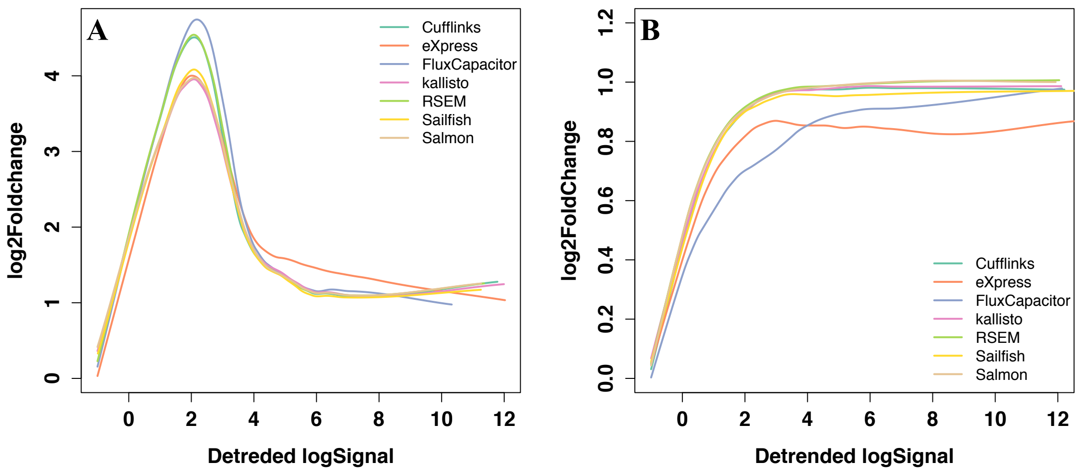


**Figure S5.** Log fold changes of true differential expressions fitted by loess. (A) Plot is based on experimental dataset with cell lines GM12878 and K562. True differential genes are estimated by microarray data. (B) Plot is based on simulation dataset with true differential transcripts predefined.

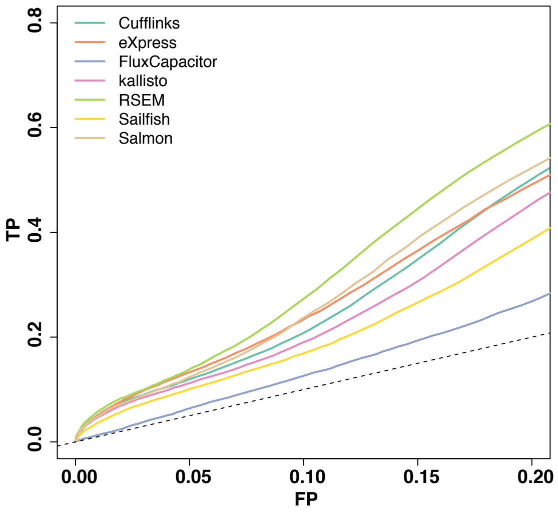


**Figure S6.** ROC curves on simulation dataset when no filtering is applied on expression calls.
