# Supplementary figures and images for: Erratum to: A benchmark for RNA-seq quantification pipelines
Source: Genome Biol. 2016 Sep 30;17:203. doi: 10.1186/s13059-016-1060-7 (PMC5045616; doi:10.1186/s13059-016-1060-7)

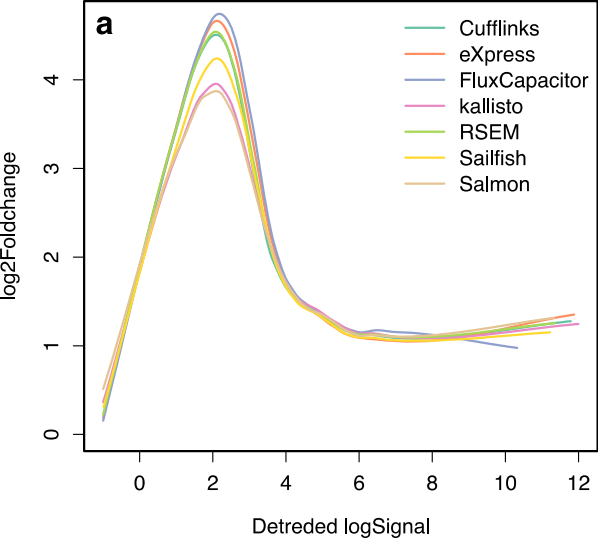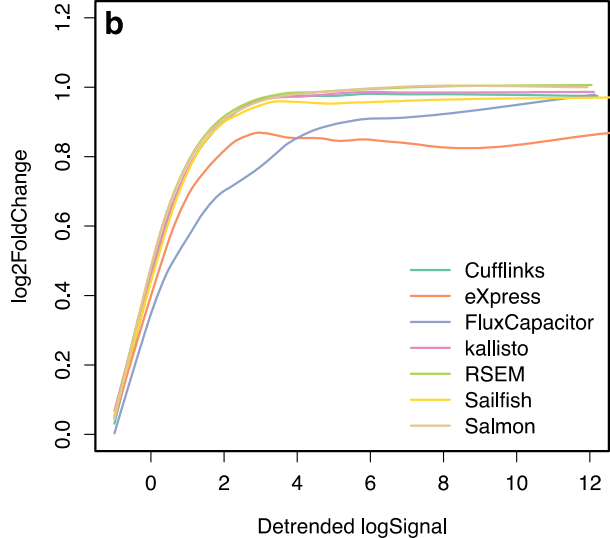

Supplement: Additional file 1: Figure S5. — Log fold changes of true differential expression fitted by losses. (a) Plot based on experimental dataset from cell lines GM12878 and K562. True differentially expressed genes are estimated using microarray data. (b) Plot based on simulation dataset with true differentially expressed transcripts predefined. (PDF 100 kb) [file 13059_2016_1060_MOESM1_ESM.pdf]
